# Most postoperative reserved “normal” metatarsal stumps of diabetic foot osteomyelitis are infected but have healing potential

**DOI:** 10.3389/fendo.2023.1165305

**Published:** 2023-08-02

**Authors:** Jun Xu, Weiling Chen, Lu He, Shuhong Feng, Jinghang Zhang, Bai Chang

**Affiliations:** ^1^ National Health Commission of the People’s Republic of China Key Laboratory of Hormones and Development, Tianjin Key Laboratory of Metabolic Diseases, Department of Diabetic Foot, Chu Hsien-I Memorial Hospital and Tianjin Institute of Endocrinology, Tianjin Medical University, Tianjin, China; ^2^ Department of Dermatology, Dongzhimen Hospital of Beijing University of Traditional Chinese Medicine, Beijing, China; ^3^ National Health Commission of the People’s Republic of China Key Laboratory of Hormones and Development, Tianjin Key Laboratory of Metabolic Diseases, Department of Clinical Laboratory, Chu Hsien-I Memorial Hospital and Tianjin Institute of Endocrinology, Tianjin Medical University, Tianjin, China

**Keywords:** diabetic foot osteomyelitis, reserved metatarsal stump, infection, conservative surgery, wound healing

## Abstract

**Background:**

Although the pathology and bacterial status of the “normal” bone stump after operation of diabetic foot osteomyelitis (DFO) are of great significance for the prognosis of foot wounds, there are only a few studies on this topic; hence, it is clinically relevant and urgent to study this topic.

**Methods:**

The data of 57 inpatients with DFO from June 2021 to April 2022 were collected, all of whom had DFO in the forefoot and underwent conservative surgery. After the surgical removal of necrotic bone, bone biopsies were taken from the necrotic phalangeal bone and the reserved “normal” metatarsal stump. They were cultured, after which antibiotic susceptibility test and pathological screening were carried out. According to clinical judgment, inpatients’ wounds were divided into metatarsal affected group and metatarsal unaffected group. We then compared and analyzed the pathological and bacterial characteristics of preserved “normal” bone stump and its effect on wound healing and prognosis.

**Results:**

The poor concordance rate between deep soft tissue culture and infected phalange culture was only 19.3%. The deep soft tissue (72.6%), infected phalange (70.7%), and metatarsal stump (71.4%) were mainly infected with gram-negative Bacillus. The proportion of *Enterococcus spp.* increased significantly in bone tissue. *Acinetobacter baumannii* had the highest drug resistance (88%, 22/25). There was no significant difference in several clinical characteristics and wound healing regardless of whether their metatarsal stumps were affected. Most reserved “normal” metatarsal stumps (84.2%, 48/57) were positive by pathological diagnosis and bacterial culture testing; only 15.7% (9/57) samples were truly sterile. Only 8.3% (4/48) of the former patients healed within 6 months; whereas, all the latter (9/9) patients healed within 6 months. However, the majority (89.6%, 43/48) could heal. There was no difference in operations, skin grafting, negative pressure wound therapy, and mortality between the two groups.

**Conclusion:**

The most reserved “normal” metatarsal stumps have been invaded by bacteria. However, the majority stumps can be preserved, and the wound will eventually be healed according to the pathological and bacterial culture results.

## 1 Introduction

According to the diabetes map of International Diabetes Federation (10th version), there are 578 million diabetic patients in the world (1). Diabetic foot is a common chronic complication of diabetes. According to the International Working Group on Diabetic Foot (IWGDF) report, one patient will lose a leg because of diabetic foot every 20 s (2). Diabetic foot is associated with serious financial and health-related burden to the affected patients, their families, and society. Diabetic foot infection is one of the main causes for hospitalization of diabetic patients. If the infection is not treated promptly and appropriately, then it will often lead to amputation or even death (3–6). Diabetic foot osteomyelitis (DFO) has always been an important topic in clinical practice, both in diagnosis and treatment. In the past, clinicians often pointed out that infected bone should be completely removed. However, recent studies have reported that the DFO in toe can heal without amputation and with only using antibiotics (7). Further studies recommend the use of conservative surgery, in that, after complete removal of the necrotic bone, even if the adjacent bone is infected, it can be retained provided that it is “normal” after the operation and it is hard with healthy, red bone marrow and with no obvious purulent necrosis (8). The question remains whether it is necessary to conduct bone culture and pathology for the preserved “normal” stump by clinical observation? In addition, is the preserved “normal” stump truly sterile? Finally, in case of residual infection, is there any difference between the postoperative treatment and wound healing and the truly sterile stump. To our knowledge, there is a scarcity of research around these questions, and, hence, it is clinically relevant and urgent to study this topic.

## 2 Materials and methods

Patients hospitalized in the Department of Diabetic Foot Chu Hsien-I Memorial Hospital, Tianjin Medical University, from June 2021 to April 2022 were selected if they met the following criteria: (i) Patients with a diagnosis of diabetes based on the WHO criteria; (ii) those with diabetic foot who met the IWGDF guidelines and had a grading of their infectious severity; (iii) the infection was mainly in the phalanges and/or the metatarsal bones; (iv) all osteomyelitis cases were confirmed by biopsy of the affected phalangeal bone during the operation, and at least one positive bacterial culture or diagnostic bone histopathology confirmed phalangeal osteomyelitis; (v) after amputation of the infected and necrotic phalanges, the preserved metatarsal stumps were sampled by bone biopsy, including bacterial culture and histopathology; and (vi) patients voluntarily agreed to participate in this study.

According to the above inclusion criteria, 57 patients with DFO were enrolled for the final analysis.

The following exclusion criteria were applied: (i) Patients with DFO who were only treated with antibiotic treatment and simple debridement without amputation; (ii) patients with DFO in the heel; (iii) pregnant patients; and (iv) those who were unable to cooperate with the study.

All patients’ demographic characteristics; duration of diabetes and diabetic foot; and complications such as coronary heart disease, stroke, hypertension, dyslipidemia, hyperuricemia, or gout were recorded. Glycosylated hemoglobin A1c; biochemical indicators; and infectious indicators such as white blood cell count and neutrophils percentage, C-reactive protein, procalcitonin, and erythrocyte sedimentation rate were measured by the hospital laboratory. Peripheral arterial disease was diagnosed by foot artery palpation, ultrasound, or transcutaneous oxygen pressure. Diabetic peripheral neuropathy was diagnosed by a 10-g monofilament and 128-Hz tuning fork.

The enrolled patients with DFO in the forefoot, as well as those of diabetic complication, were treated with antiglycemic medicine or insulin, antibiotics, and debridement. We also provided basic treatment for hypertension, dyslipidemia, hyperfibrinogenemia, elevated D-dimer levels, and cessation of smoking. Deep tissue for bacterial culture was collected from all patients at admission. Because their toes were severely infected and necrotic and could not be preserved, they were all amputated. The management of the adjacent metatarsal bones involved two situations. One was when the metatarsals were unaffected upon clinical observation. However, after treatment of the metatarsophalangeal joint capsule, the metatarsal bones were not conducive to granulation growth because of the presence of the joint surface. The conventional method is to remove the joint surface and expose the metatarsal head, which is conducive to wound healing. The other was that the adjacent metatarsal bone was damaged and needed surgical debridement until the surgeon deemed the reserved metatarsal stump as being “normal.” All infected phalanges and the reserved “normal” metatarsal stump were subjected to bone biopsy. The former was to determine the diagnosis of osteomyelitis, and the latter was to determine whether there was infection and whether the infected stump had an impact on wound healing. The use of antibiotics was guided by the results of bone culture and the reaction of the wound after the operation. The treatment of negative pressure drainage, various growth factors, and skin grafting was carried out according to the situation of the wound (9).

All patients were followed up at the diabetic foot outpatient clinic for at least 6 months after discharge. On average, patients came to the outpatient clinic for a follow-up visit every 2–4 weeks based on condition of their wounds.

The first author and the corresponding author completed all the surgical procedures of this study; the third author completed all the bacterial cultures; and a pathologist from the Department of Pathology interpreted all histopathological findings. This study was approved by the institutional ethics committee, and all patients signed the informed consent form.

SPSS 28.01.1 (IBM Corporation, Armonk, NY, USA) was used for data analysis. Levene test was used for normality testing of quantitative data. F-test or t-test was used for normally distributed quantitative data, and Mann–Whitney U-test was used for non-normally distributed quantitative data. Qualitative data were compared using χ2 test, but, if the expected value of the cell was <5, then Fisher’s exact test was used. P < 0.05 was considered to indicate statistically significant differences.

## 3 Results

### 3.1 Bacterial composition of diabetic foot wound

**Figure 1:**
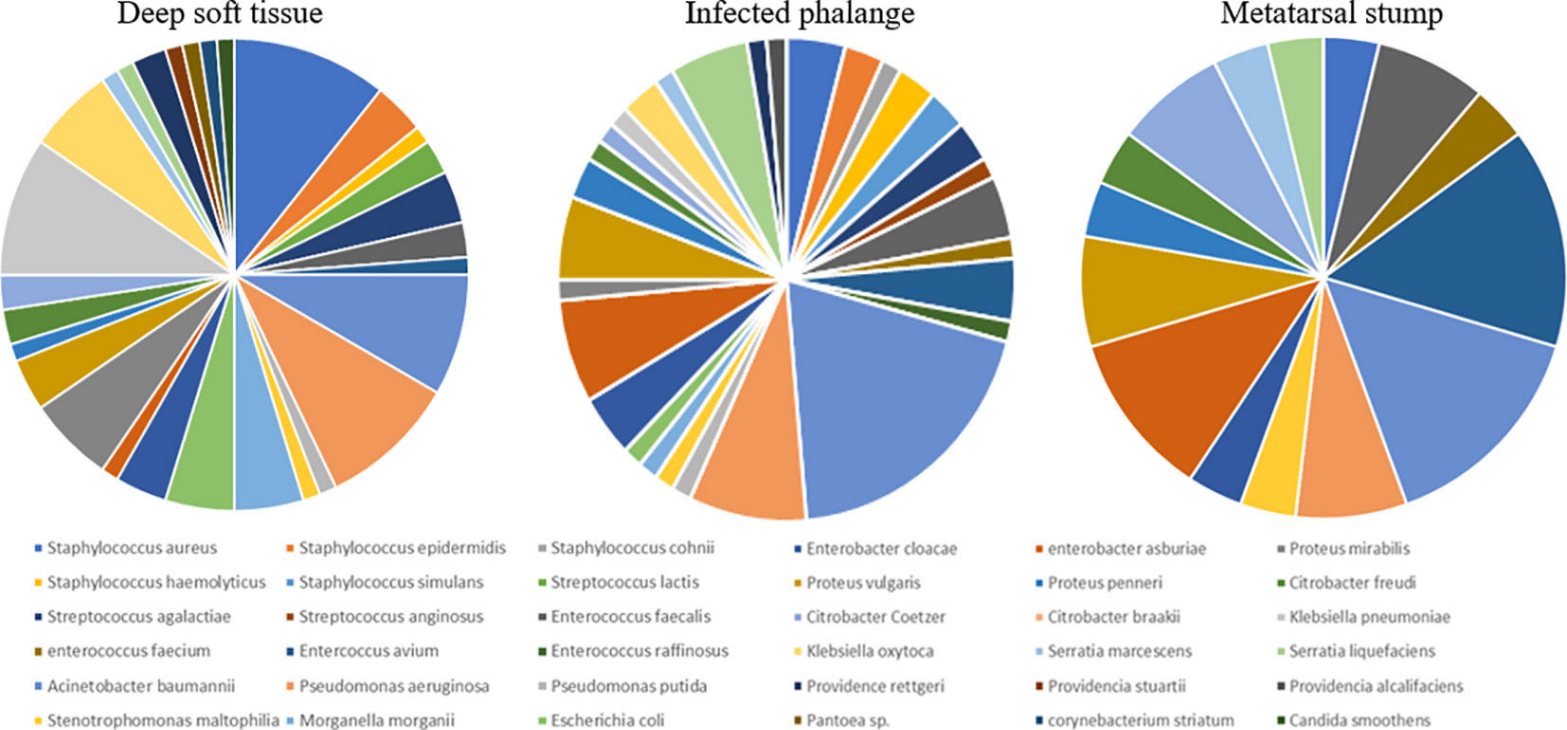
The composition of bacteria in deep soft tissue, infected phalange, and “normal” reserved metatarsal stump. Different colors represented different bacteria. The larger was the share, the more was the number of bacteria in the corresponding group. At least 28 species of bacteria had been cultivated from the three kinds of tissue samples. In addition, the specific proportion of bacteria was different, but most of them were Gram-negative bacillus, all were more than 70%.

In the foot wounds of 57 patients, one fungus and 83 bacteria were cultured in the deep soft tissue, one fungus and 74 bacteria were cultured in the phalanges, and 28 bacteria were cultured in the metatarsal stumps. The composition of specific bacteria is presented in **Figure 1**.

Among the deep soft tissue, gram-positive cocci accounted for 25%, gram-negative bacilli accounted for 72.6%, and one gram-positive bacilli (*Corynebacterium striatum*) and one fungus (*Candida smoothens*) accounted for 1.2%, respectively. The top three bacteria were *Staphylococcus aureus* (nine strains), *Klebsiella pneumoniae* (eight strains), and *Pseudomonas aeruginosa* (eight strains). Among the gram-positive cocci, the first three were *Staphylococcus aureus* (nine strains), *Streptococcus lactis* (three strains), and *Staphylococcus epistaphylum* (three strains). Among gram-negative bacilli, the first three were *K. pneumoniae* (eight strains), *P. aeruginosa* (eight strains), and *Acinetobacter baumannii* (seven strains). In the phalanges, gram-positive cocci accounted for 28%, gram-negative bacilli accounted for 70.6%, and one fungus accounted for 1.3%. The top three bacteria *were A. baumannii* (14 strains), *P. aeruginosa* (six strains), and *Enterobacter cloacae* (five strains). Among gram-positive cocci, the top three were *Staphylococcus aureus* (three strains), *Enterococcus faecalis* (three strains), and *Enterococcus avium* (three strains). The sequence of gram-negative bacilli was the same as the total sequence. In the metatarsal stumps, gram-positive cocci accounted for 28.6%, and gram-negative bacilli accounted for 71.4%. The top three bacteria were *A. baumannii* (four strains), *Enterococcus avium* (four strains), and *Enterobacter cloacae* (three strains). Among the gram-positive cocci, the first three were *Enterococcus avium* (four strains), *Enterococcus faecalis* (four strains), *Staphylococcus aureus* (one strain), and *Enterococcus faecium* (one strain). Among the gram-negative bacilli, *A. baumannii* (four strains), *Enterobacter cloacae* (three strains), and two strains each of *P. aeruginosa*, *Citrobacter freundii*, and *Proteus mirabilis* were found.

From the above bacterial distribution, the following results can be obtained: Deep soft tissue or bone tissue was mainly infected with gram-negative bacilli.

### 3.2 Distribution of antibiotics resistant bacteria

The antibiotic resistant rate of deep soft tissue, phalange, and metatarsal stump was not statistically significant (all P > 0.05) (**Table 1**). Overall, *A. baumannii* had the highest antibiotic resistance (88%, 22/25)

**Table 1:** Distribution of antibiotic resistant bacteria.

	Deep soft tissue				Infected phalange				Metatarsal stump			
	n	First	Second	Third	n	First	Second	Third	n	First	Second	Third
G (+)	1	MRSA1			1	MRSA1			0			
G (−)	10	CRAB6	*Escherichia coli* ESBL 1, *Klebsiella pneumoniae* ESBL 1, *Proteus mirabilis*, CRE 1, *Proteus vulgaris* CRE 1		18	CRAB 13	*Proteus mirabilis* CRE2	*Escherichia coli*, ESBL 1, CRPA 1, *Proteus vulgaris* CRE 1	5	CRAB3	*Proteus mirabilis* CRE 1, *Proteus vulgaris* CRE 1	
Gram (+) antibiotic resistance rate	4.8% (1/21)				4.8% (1/21)				0			
Gram (−) antibiotic resistance rate	16.4% (10/61)				34.0% (18/53)				25% (5/20)			
Total Antibiotic resistance rate	13.4% (11/82)				25.7% (19/74				17.9% (5/28)			

The antibiotic resistance rate in deep soft tissue, infected phalangeal, and metatarsal stump was no statistical significance (all P > 0.05).

CRE, carbapenem-resistant Enterobacteriaceae; ESBL, extended-spectrum beta-lactamase; MASR, methicillin-resistant Staphylococcus aureus.

G (+), gram positive coccus; G (−), gram negative bacilli.

### 3.3 Distribution of bacterial species

**Figure 2:**
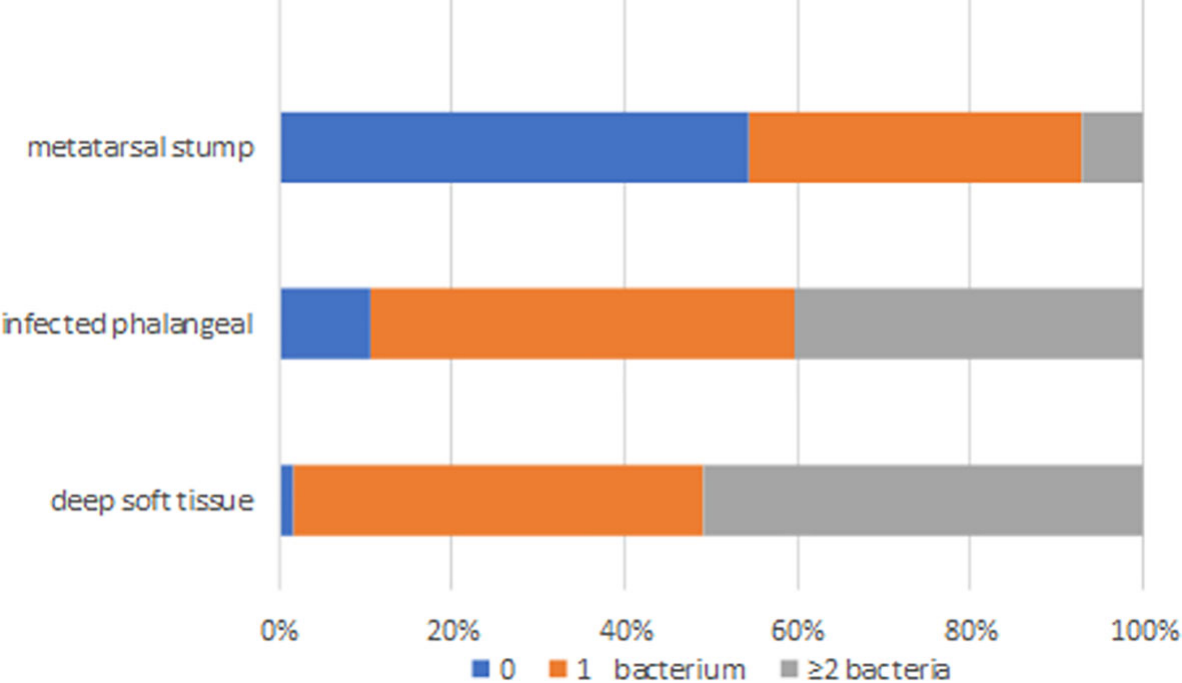
The distribution of bacteria species in deep soft tissue, infected phalange, and “normal” reserved metatarsal stump. 0 represented sterile, 1 represents one kind of bacteria, and ≥ 2 represented two or more different kinds of bacteria in sample.

The distribution of bacterial species in different tissues is shown in **Figure 2**. Only 54.4% of the clinically preserved “normal” metatarsal stumps were sterile.

### 3.4 Concordance of bacteria culture from deep soft tissues, phalanges and metatarsal stumps

#### 3.4.1 Concordance of bacteria culture from deep soft tissues and phalanges

The concordance of isolated bacteria between the deep soft tissues and phalanges was low, and only 10 wounds (17.5%) had the same bacteria. The proportion of Enterococcus spp. in bone increased, and most of the corresponding soft tissue did not exist (**Table 2**).

**Table 2:** Concordance of bacteria in deep soft tissues and phalanges.

No. 57	Concordance	Difference 47		Bone negative
		Completely difference	Partial difference	
	10	19	23	5
	(17.5%)			

Completely different meant that there were no same bacteria between the wound deep soft tissue and phalange. Partial difference meant there was at least one species of bacteria consistent between deep soft tissue and phalange. Bone negative meant phalangeal bone culture was negative (but DFO was still established, and its pathology was positive).

#### 3.4.2 Concordance of bacteria culture from phalanges and metatarsal stump

Although all the reserved metatarsal stumps in the operation were considered “normal,” there were two situations requiring different management: In one, the metatarsal bone was unaffected, whereas, in the other, the metatarsal bone was affected. The patients were divided into two groups for comparison of the clinical outcomes.

**Table 3** shows that, even if the infected and necrotic part of the affected metatarsal bone was removed in the operation, the negative rate of the retained “normal” metatarsal stump was only 42.4% (14/33). Only 24.2% of the metatarsal and phalangeal bacteria were completely consistent, and bacteria changed in 33% of all metatarsal stumps. Although the unaffected metatarsal bone group was considered be sterile, there was only 62.5% sterility. The metatarsal stump still had bacteria, but it was consistent with the phalange. However, there was no statistically significant difference in the distribution of bacteria between the two groups (Fisher’s exact test: 4.822, P = 0.155).

**Table 3:** Concordance of bacteria in phalanges and metatarsal stumps.

	Concordance	Difference		Bone negative
		Total difference	Partial difference	
Affected metatarsal “normal” stumps	8	2	9	14
33				
Unaffected metatarsal “normal” stumps	7	0	2	15
24				

### 3.5 Analysis of metatarsal stumps

**Table 4** shows that the proportion of osteomyelitis in the affected vs. unaffected metatarsal groups was 60.6% (20/33) vs. 29.2% (7/24), respectively, only by bacterial culture diagnosis, and the proportion of osteomyelitis was 93.9% (31/33) vs. 62.5% (15/24), respectively, only by pathological diagnosis. The differences were statistically significant (all P < 0.05). The positive rate of pathology was significantly higher than that of bacterial culture, but it did not reach 100%.

**Table 4:** Analysis of metatarsal stump.

	No.	Bacterial culture (+)	Pathology (+)	Bacterial culture + Pathology (+)	Bacterial culture + Pathology (−)	Metatarsal bone positive by X ray	Bacterial culture (−)	Pathology (−)
Clinically affected metatarsal group	33	1	12	19	1	20	13	2
Clinically unaffected metatarsal group	24	1	9	6	8	0	17	9
Fisher’s exact test	11.592					–	5.509	–
P-value	0.004					0.003	0.019	0.005

(+) meaning positive. (-) meaning positive. The results of Fisher's exact test only have P value; -, do not have statistics value.

### 3.6 Clinical characteristics and prognosis

#### 3.6.1 Clinical characteristics

**Table 5** shows that the procalcitonin level of patients with affected metatarsal was higher than that of patients with unaffected metatarsal. The positive rate of X-ray was higher in the affected metatarsal group than that in the unaffected metatarsal group. There was no statistical difference between other indicators.

Table 5Clinical characteristics between clinically affected and unaffected metatarsal groups.

**Table:** 

		No.	Gender (m/f)		Age (year)	Duration of diabetes (years)	HbA1c (%)	Duration of diabetes (months)	Severity of infection (moderate/severe)	CRP (mg/L)	ESR (mm/h)	Procalcitonin (>0.05 μg/L positive)	
Clinically affected metatarsal group		33	22/11		62.7 ± 10.6	12.7 ± 8.4	8.2 ± 2.3	2.89 ± 3.16	20/13	67.6 ± 78.3	48.2 ± 17.4	7/26	
Clinically unaffected metatarsal		24	16/8		65.5 ± 9.1	14.5 ± 9.2	7.9 ± 1.9	1.97 ± 2.27	20/4	53.9 ± 67.4	48.0 ± 15.4	0/24	
Statistics			0.0		−1.030	−0.758	0.46	1.419	3.429	0.689	0.053	–	
P-value			1.0		0.307	0.452	0.647	0.162	0.064	0.494	0.958	0.017

**Table:** 

	WBC (×10^9^/L)	Neutrophils (%)			IWGDF infection severity grade (moderate/severe)	TcPO_2_ (mmHg)	eGFR (ml/min)	Fibrinogen (g/L)	D-dimer (mg/L)		Albumin (g/L)	hemoglobin (g/L)	DR (±)
Clinically affected metatarsal group	12.55 ± 8.42	73.81 ± 9.92			15/18	24.5 ± 18.6	82.4 ± 28.5	6.07 ± 1.90	1.119 ± 0.89		32.3 ± 5.2	100.0 ± 24.5	23/10
Clinically unaffected metatarsal	9.66 ± 6.64	71.45 ± 10.08			17/7	28.8 ± 17.4	80.4 ± 20.1	5.43 ± 1.76	1.27 ± 1.10		33.5 ± 2.9.0	109.4 ± 22.4	18/6
Statistics	1.395	0.801			3.635	−0.891	0.414	1.28	−0.568		−1.057	−1.473	0.194
P-value	0.169	0.426			0.057	0.377	0.68	0.206	0.572		0.295	0.146	0.660

**Table:** 

		CHD (±)		Stroke (±)	Hypertension (±)		Dyslipidemia (±)	Hyperuricemia or gout (±)			Probe to bone test (±)	X ray (soft tissue+/phalange +/metatarsal bone+)	
Clinically affected metatarsal group		18/15		12/21	17/16		20/13	6/27			28/5	5/9/20	
Clinically unaffected metatarsal group		15/9		11/13	17/7		15/9	5/19			19/5	6/17/0	
Statistics		0.361		0.518	2.154		0.021	–			–	26.858	
P-value		0.548		0.472	0.142		0.885	1.000			0.727	0.000	

HbA1c, glycated hemoglobin A1c; CRP, C-reactive protein; ESR, erythrocyte sedimentation rate; WBC, white blood cell; TcPO_2_, transcutaneous oxygen pressure; eGFR, estimated glomerular filtration rate; DR, diabetic retinopathy; CHD, coronary heart disease. The results of Fisher's exact test only have P value; -, do not have statistics value.

#### 3.6.2 Comparison of operation and prognosis

It can be seen from **Table 6** that there was no statistical difference in wound healing, mortality, amputation, and negative pressure use, but patients in the group with clinically affected metatarsal needed more skin grafts than those in the group with clinically unaffected metatarsal (P = 0.007). Three patients died because of poor control of foot infection, difficult wound healing, and heart and multi-organ failure, all of which were related to the foot infection.

**Table 6:** Operation and prognosis between clinically affected and unaffected metatarsal groups.

	Wound healing					death	Amputation			Negative pressure wound therapy	Skin grafting
	Healing time < 6 months	Healing time ≥ 6 months	Unhealing	Recurrence	New wound		Primary minor amputation1	Secondary minor amputation	Major amputation		
Clinically affected metatarsal group	7	20	3	2	1	1	26	4	3	21	9
Clinically unaffected metatarsal group	6	12	2	1	3	2	21	3	0	15	0
Statistics	2.385					–	1.977			0.008	–
P-value	0.716					0.567	0.418			0.93	0.007

The results of Fisher's exact test only have P value; -, do not have statistics value.

### 3.7 Clinical characteristics and outcomes of patients with true metatarsal negative

#### 3.7.1 Clinical characteristics

There were positive (infected) metatarsal stumps (84.2%, 48/57) in the reserved “normal” metatarsals stump based on surgical diagnosis, regardless of whether the metatarsal was affected on clinical observation. However, there were also true sterile stumps with negative pathology and bacteria. There were eight patients in the clinically unaffected metatarsal group and one in the clinically affected metatarsal group. **Table 7** shows the comparison of differences in clinical characteristics and prognosis between these nine patients with true negative metatarsal stumps and other patients.

Table 7Clinical characteristics between patients with true negative metatarsal stump with others.

**Table:** 

	No.	Gender (m/f)	Age (years)	Duration of diabetes (years)	HbA1c (%)	Duration of diabetes (months)	Severity of infection (moderate/severe)	CRP (mg/L)	ESR (mm/h)	Procalcitonin (>0.05 μg/L positive)
Patients with true negative metatarsal stump	9	5/4	61.0 ± 10.3	13.4 ± 8.5	7.5 ± 1.5	1.8 ± 1.7	7/2	50.7 ± 53.1	47.1 ± 18.2	0/9
others	48	33/15	64.4 ± 10.0	13.5 ± 8.9	8.2 ± 2.2	2.7 ± 3.0	33/15	66.9 ± 77.1	48.3 ± 16.3	7/41
Statistics		–	0.934	0.009	0.906	0.781	–	0.490	0.192	–
P-value		0.463	0.354	0.993	0.369	0.438	0.71	0.626	0.848	0.582

**Table:** 

	WBC (×10^9^/L)	Neutrophils (%)		TcPO_2_ (mmHg)	eGFR (ml/min)	Fibrinogen (g/L)	D-dimer (mg/L)		Albumin (g/L)	Hemoglobin (g/L)
Patients with true negative metatarsal stump	8.11 ± 2.44	68.98 ± 9.35		28.3 ± 15.8	75.9 ± 21.8	5.54 ± 2.26	1.35 ± 1.61		34.6 ± 3.2	110.4 ± 31.0
Others	11.94 ± 8.31	73.53 ± 9.88		25.9 ± 18.6	82.6 ± 25.8	5.85 ± 1.79	1.15 ± 0.84		32.5 ± 4.5	102.7 ± 22.5
Statistics	1.362	1.28		−0.369	0.730	0.457	−0.57		−1.341	−0.886
P-value	0.179	0.206		0.714	0.468	0.649	0.571		0.185	0.38

**Table:** 

	CHD (±)	Stroke (±)		Hypertension (±)	Dyslipidemia (±)	Hyperuricemia or gout (±)	Probe to bone test (±)		DR (±)	
Patients with true negative metatarsal stump	7/2	4/5		8/1	5/4	2/7	7/2		7/2	
Others	26/22	19/29		26/22	30/18	9/39	40/8		34/14	
Statistics	–	–		–	–	–	–		26.858	
P-value	0.277	1.0		0.069	0.722	1.0	0.65		1.0	

HbA1c, glycated hemoglobin A1c; CRP, C-reactive protein; ESR, erythrocyte sedimentation rate; WBC, white blood cell; TcPO_2_, transcutaneous oxygen pressure; eGFR, estimated glomerular filtration rate; DR, diabetic retinopathy; CHD, coronary heart disease. The results of Fisher's exact test only have P value; -, do not have statistics value.

There was no difference in clinical characteristics between the two parts from **Table 7**.

#### 3.7.2 Outcome and prognosis

We found that the nine patients healed faster than other patients (P < 0.05) (**Table 8**). There was no difference in surgical procedure, skin grafting, negative pressure use, and mortality.

**Table 8:** Operation and prognosis between patients with true negative metatarsal stump with others.

	No.	Wound healing					Death	Amputation			Negative pressure wound therapy	Skin grafting
		Healing time < 6 months	Healing time ≥ 6 months	unhealing	recurrence	New wound		Primary minor amputation	Secondary minor amputation	Major amputation		
Patients with true negative metatarsal stump	9	9	0	0	0	0	0	9	0	0	3/6	0
Others	48	4	32	5	3	4	3	38	7	3	33/15	9
Fisher’s exact test		27.333					–	1.283			–	–
P-value		0.000					1.0	0.755			0.063	0.328

The results of Fisher's exact test only have P value; -, do not have statistics value.

## 4 Discussion

Given the increasing incidence of diabetes worldwide, the number of patients with diabetic foot is also increasing year by year. More than 50% patients with diabetic foot are further complicated with infections (10, 11). Typically, patients with DFO need urgent medical treatment or even hospitalization. Given the complexity of treating DFO, the IWGDF infection guidelines (2019 edition) have introduced some changes, in that, in the case of moderate and severe infection and if osteomyelitis exists, osteomyelitis must be specifically marked “O” followed by moderate or severe infection (2). This is because the diagnosis and treatment of DFO are more difficult than that of soft tissue infection.

The treatment of DFO has always been a hot topic of research and debate. In recent years, some studies have suggested that osteomyelitis of the forefoot is caused by only neuropathy with good blood supply, provided that there is no serious destruction of the joint capsule, rather only bone exposure. DFO can heal through antibiotic treatment and appropriate debridement, and toe amputation can be avoided (12, 13). However, in clinical practice, the proportion of such patients is limited. Although 90% of osteomyelitis occurs in the forefoot, in many cases, the infection range is large, as the phalangeal bone is broken, and the metatarsophalangeal joint capsule is damaged. Hence, toe amputation is often required. Understandably, the optimum approach to treat has been controversial. If the metatarsal bone is damaged and purulent, then it will be removed, but, generally, the entire metatarsal bone will not be taken off (unless the entire metatarsal bone is damaged and the infection extends to the midfoot or even the hindfoot). Instead, it will be retained up to the residual end of the metatarsal bone that the surgeon considers “normal.” However, it is impossible to immediately determine whether these stumps are truly sterile based on pathology or bacteria culture. The IWGDF guidelines state that the relationship between the true infection of these “normal” stumps and prognosis should be urgently studied. Clinically, for cases where the phalangeal bone is completely necrotic, part of the metatarsophalangeal joint capsule is involved, but the side connected with the metatarsal bone is normal are also encountered, but these conditions are not conducive to granulation growth and wound healing. For the purpose of promoting growth, experts have reached a consensus that it is necessary to open the joint capsule and expose the metatarsal head to facilitate the growth of granulation tissue and accelerate wound healing (14–16). However, it is yet unclear whether this part of the metatarsus is affected.

All 57 patients selected in this study had osteomyelitis of the forefoot, and the diagnosis was confirmed by intraoperative bone biopsy. We found that the proportion of gram-negative bacilli was significantly higher than that of gram-positive cocci in the soft tissue and bone tissue (all >70%). The prevalence of gram-positive cocci (43.4%) was lower than that of gram-negative bacilli (52.4%) in China (17). In terms of antibiotic-resistant bacteria, we found *A. baumannii* had the highest drug resistance rate. Enterococcus spp. had more infection in bone, and there was no infection in the corresponding soft tissue. Because the choice of antibiotics for Enterococcus spp. has certain characteristics, more attention should be paid to it in clinical practice.

The consistency rate of bacteria cultured in soft tissue and bone tissue was very low, <20% in this study. Senneville et al. reported the overall concordance was 22.5% (18). Because our diabetic foot centers are in a tertiary-care hospital, most patients used antibiotics, and we did not stop antibiotic use before collecting samples. All patients in this study had osteomyelitis of the phalanges. On the basis of clinical judgment, patients were divided into the affected metatarsal group and unaffected metatarsal group. However, the metatarsal bone sample that we obtained was the “normal” stump per clinical judgment. These reserved stumps were not truly sterile. Even in the unaffected group, 37.5% samples tested positive for bacterial growth, but the consistency with the phalanges was good. In the affected metatarsal group, only 42.4% stumps were truly sterile, despite being judged as “normal” by the surgeons. Moreover, the bacteria in the metatarsal bone were not consistent with those in the phalangeal bone, which merits further investigation. It should be noted that the infection rate of the retained metatarsal stump judged by pathology and bacterial culture was <100%, although the positive rate of pathology was higher than that of bacterial culture. Hence, it is recommended to carry out both tests at the same time.

We found that there was no significant difference in many clinical characteristics between the two groups regardless of whether their metatarsal bones were involved before the operation. On the one hand, the cardiovascular risk factors such as hypertension, diabetes, dyslipidemia, smoking, hyper D-dimer, and hyperfibrinogenemia and cardiovascular events such as coronary heart disease and stroke existed in both groups and showed no differences. We prescribed standard, routine treatment as needed. Some patients with acute myocardial infarction, heart failure, and stroke had a poor wound healing, including bone healing, but there was no difference between the two groups. On the other hand, patients with cardiovascular risk factors were given comprehensive treatment, which would not occur the cardiovascular events, and, then, the wound healing and bone healing were better. There were more procalcitonin-positive patients in the affected group than that in unaffected metatarsal group. In addition, the affected metatarsal group had a higher number of X-ray findings than the unaffected metatarsal group. After surgical treatment, there was no difference between the two groups in terms of wound healing, minor amputation times, death, major amputation, and negative pressure use. The affected metatarsal group needed more skin grafts than the unaffected group, which was related to the large wound size. On the basis of these findings, we point out that the surgical methods used in this study were effective for the above two groups of patients.

Among these patients, only nine patients’ metatarsal stumps were negative by both pathology and bacterial culture. These patients had true normal metatarsal stumps, only accounting for 15.8% (9/57). Unfortunately, there was no significant clinical difference between them and other patients. Eight of these patients were from the unaffected metatarsal group and one from the affected metatarsal group. The only difference between these nine patients and other patients was that the healing time was fast, as they all healed within 6 months. The other 40 patients (excluding the three deaths) finally healed. The causes of three deceased patients were myocardial infarction (two patients) and COVID-19 (one patient). The main reason for the five of the 48 patients who did not heal was the presence of severe peripheral arterial disease and the inability or failure of revascularization.

This study has the following strengths: First, our diabetic foot center has 60 beds in the ward, all of which are used to treat patients with diabetic foot. The researchers are all diabetic foot professionals. Two surgeons (the first author and corresponding author of this study) completed the operation in and collected the bone samples from all 57 patients. There was no operating or sampling error. Second, to clarify the bacterial condition of soft tissue, samples were taken from phalangeal bone tissue and the retained metatarsal stump in diabetic foot wounds; additionally, the bone tissue was biopsied for pathological evaluation. All 57 phalangeal osteomyelitis cases were diagnosed on the basis of the gold standard technique. Third, the fifth key controversy in the IWGDF infection guideline (2019) was: “In diabetic foot osteomyelitis cases, is obtaining a specimen of residual or marginal bone after surgical resection useful for deciding which patients need further antibiotic or surgical treatment?” The residual stump that clinically is considered not to be affected has a certain proportion of infection. Antibiotic and surgical debridement should be carried out for these infected stumps. Only a small part of patients needed secondary minor amputation; most wounds patients eventually healed.

This study also has some limitations. First, this study had a single-center design. Although the 57 patients exceeded Tianjin, including some other cities, the number of cases is relatively small. Second, because the researchers work in tertiary hospitals, most patients often had a history of antibiotic use in the early stage, so the sampling was affected to some degree. Third, this study did not adopt percutaneous bone biopsy, rather used the method of intraoperative sampling.

In conclusion, we studied the characteristics of bacteria in the wound of patients with DFO. On the basis of the need for healing, the metatarsal bone was treated until the “normal stump” was exposed, regardless of whether the metatarsal bone was affected. It was confirmed that most of the retained “normal” stumps showed bacterial growth (84.2%), but these “normal” stumps can heal after treatment. The true sterile stumps (n = 9) healed quickly. Therefore, we should pay attention to the retained bone stumps, which is helpful for subsequent treatment decisions.

## Data availability statement

The raw data supporting the conclusions of this article will be made available by the authors, without undue reservation.

## Ethics statement

The studies involving human participants were reviewed and approved by Medical Ethics Committee of Chu Hsien-I Memorial Hospital Tianjin Medical University. The patients/participants provided their written informed consent to participate in this study. Written informed consent was obtained from the individual(s) for the publication of any potentially identifiable images or data included in this article.

## Author contributions

Conception and design: JX and BC. Collection and assembly of data: JX, LH, SF, and JZ. Data analysis and interpretation: SF and JZ. Manuscript writing: JX. Final approval of manuscript: All authors. All authors contributed to the article and approved the submitted version.
